# Double Axillary Vein During Axillary Lymph Node Dissection for Breast Cancer

**DOI:** 10.1002/ccr3.73139

**Published:** 2026-08-04

**Authors:** Ioannis K. Papapanagiotou, Theodoros Mariolis Sapsakos, Apostolos Mitrousias, Kyriaki Migkli, Aikaterini Giannakaki, Eftychia Papachatzopoulou, Sofia Koura, Dimitrios Papageorgiou, Evangelos Solakis, Vasileios Kalles

**Affiliations:** ^1^ Breast Department Mediterraneo Hospital Athens Greece; ^2^ Laboratory of AnatomyAdvanced Anatomical Applications of Artificial Intelligence and Experimental Surgical Research Department of Nursing, National and Kapodistrian University of Athens Athens Greece; ^3^ Surgery Department 251 Hospital of Airforce Athens Greece; ^4^ 1st Department of Obstetrics and Gynecology University of Athens Athens Greece

**Keywords:** axillary dissection, axillary lymph node, axillary vein, breast cancer, lumpectomy

## Abstract

Clinical significance of Double axillary vein is often minimal; however, it can be of concern in surgical procedures, particularly those involving axillary dissection. Injury to one of the veins could lead to severe bleeding or venous congestion, complicating the surgical outcome. Therefore, surgeons should be aware of this variation.

A 58 year old woman presented to our Breast Department with a palpable mass on her left breast. Mammography and breast ultrasound revealed an irregular mass with a maximum diameter of 1.9 cm, characterized as a BIRADS 4A lesion by 2 experienced and breast dedicated radiologists. Clinical examination as well as axillary ultrasound and breast magnetic resonance imaging did not reveal any pathological axillary nodes. Histopathology after core needle biopsy specimen retrieval indicated an invasive ductal carcinoma of the left breast. The patient underwent lumpectomy and sentinel lymph node (SLN) biopsy of the left axilla. Intraoperative frozen section of the 3 SLNs was positive for metastasis, and thus additional conventional axillary lymph node dissection was performed. At axillary dissection, a double axillary vein was detected (Figure 1). Written informed consent was obtained from the patient. None of the remaining non SLNs was metastatic at final histology.

**FIGURE 1:**
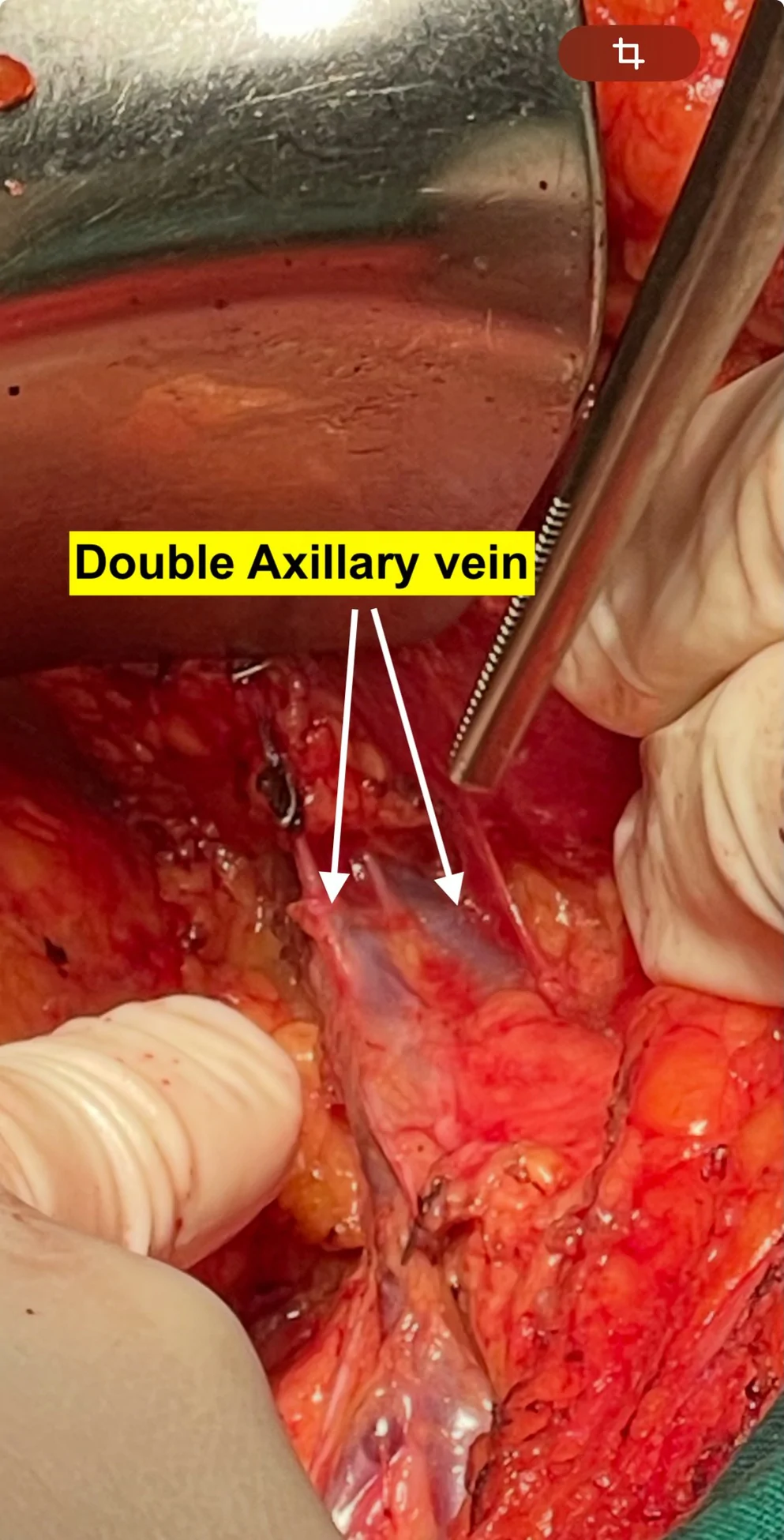
Double axillary vein of the left axilla.

The axillary vein is a large blood vessel that conveys blood from the superolateral chest wall, axilla and upper limb toward the heart. It originates from the union of the basilic vein and the brachial vein [1], usually around the inferior border of the teres major muscle. At its terminal part, it is also joined by the cephalic vein [1]. The axillary vein passes through the axilla and continues as the subclavian vein at the lateral border of the first rib. Anatomically, it lies medial to the axillary artery and is accompanied by the brachial plexus. A variant of the axillary vein, known as the double axillary vein (DAV), is characterized by the presence of two separate veins running parallel to each other, typically on either side of the axillary artery. This anatomical variation is uncommon, with an incidence rate reported to be up to 14% of patients [2]. Although the clinical significance of DAV is often minimal, it can be of concern in surgical procedures, particularly those involving axillary dissection, venous catheterization, lymph node removal or brachial plexus anesthesia [3]. In such procedures, inadvertent injury to one of the veins could lead to severe bleeding or venous congestion, complicating the surgical outcome. Additionally, the presence of a DAV may affect the placement of central venous catheters, making it more challenging to access the vein and requiring careful imaging guidance.

A comprehensive knowledge of the anatomy of the axillary region is essential for surgeons, as careful identification and preservation of key anatomical structures such as the axillary vein and the thoracodorsal bundle (TB) are required. During ALND, the axillary vein should be exposed early by removing fatty tissue from the apex of the axilla and then followed along its inferior border. Venous variants, such as duplicated or accessory veins, may be encountered and should be confirmed by careful short‐segment tracing. Dissection near the vein should avoid blind clamping or diathermy. Although vascular mapping is not routinely required, Doppler ultrasound may help identify duplicated axillary veins in selected complex or re‐operative cases. Lateral thoracic vein is a consistent tributary on the anteroinferior aspect of the axillary vein and can be used as a reliable landmark, in order to identify TB.

Surgeons should be aware of the anatomy of the axillary region; it's important anatomical structures and anatomical variations, in order to reduce the likelihood of iatrogenic injury and subsequent short‐ and long‐term patient morbidity and ensure optimal outcomes.

## Author Contributions

**Ioannis K. Papapanagiotou:** conceptualization, formal analysis, investigation, methodology, writing – original draft, writing – review and editing. **Theodoros Mariolis Sapsakos:** supervision. **Apostolos Mitrousias:** data curation. **Kyriaki Migkli:** formal analysis, investigation, resources. **Aikaterini Giannakaki:** data curation, methodology. **Eftychia Papachatzopoulou:** conceptualization, resources. **Sofia Koura:** formal analysis, methodology. **Dimitrios Papageorgiou:** resources, validation. **Evangelos Solakis:** data curation. **Vasileios Kalles:** supervision.

## Funding

The authors have nothing to report.

## Data Availability

Data and metadata are available in the Enviromental Data Initiative repository at https://doi.org/10.1002/ccr3.73139.
